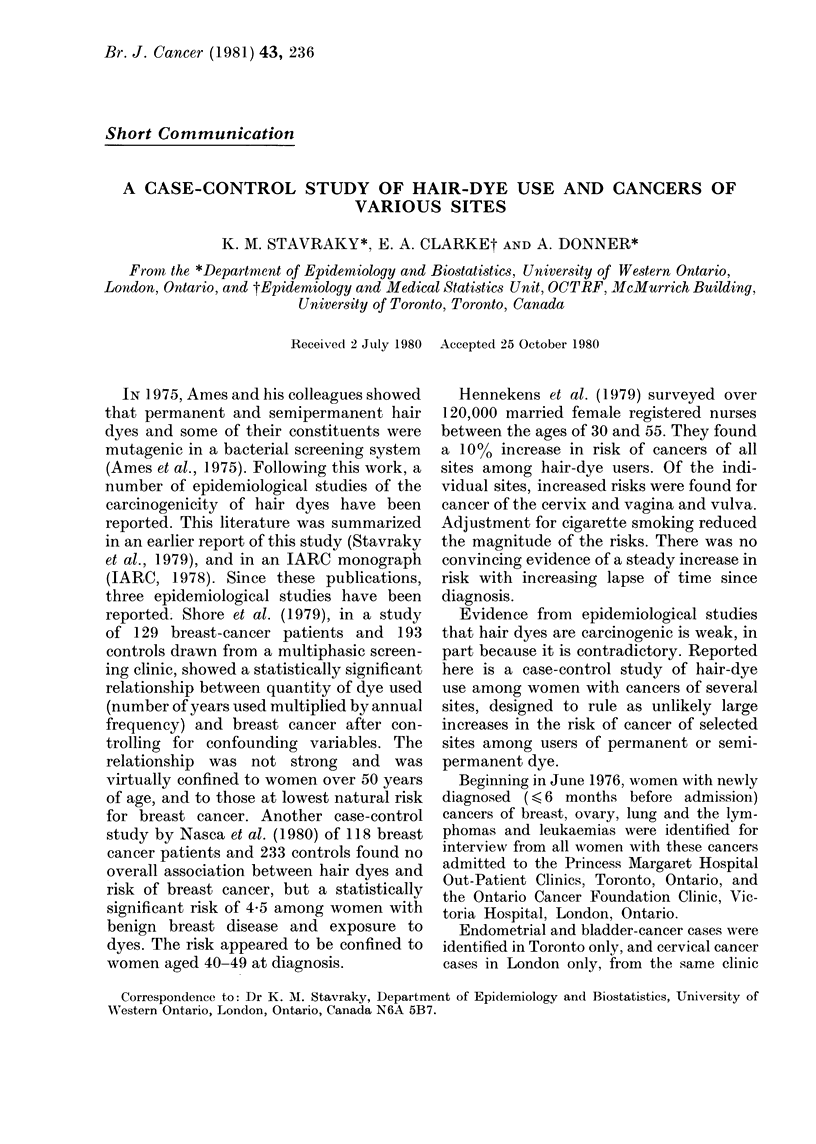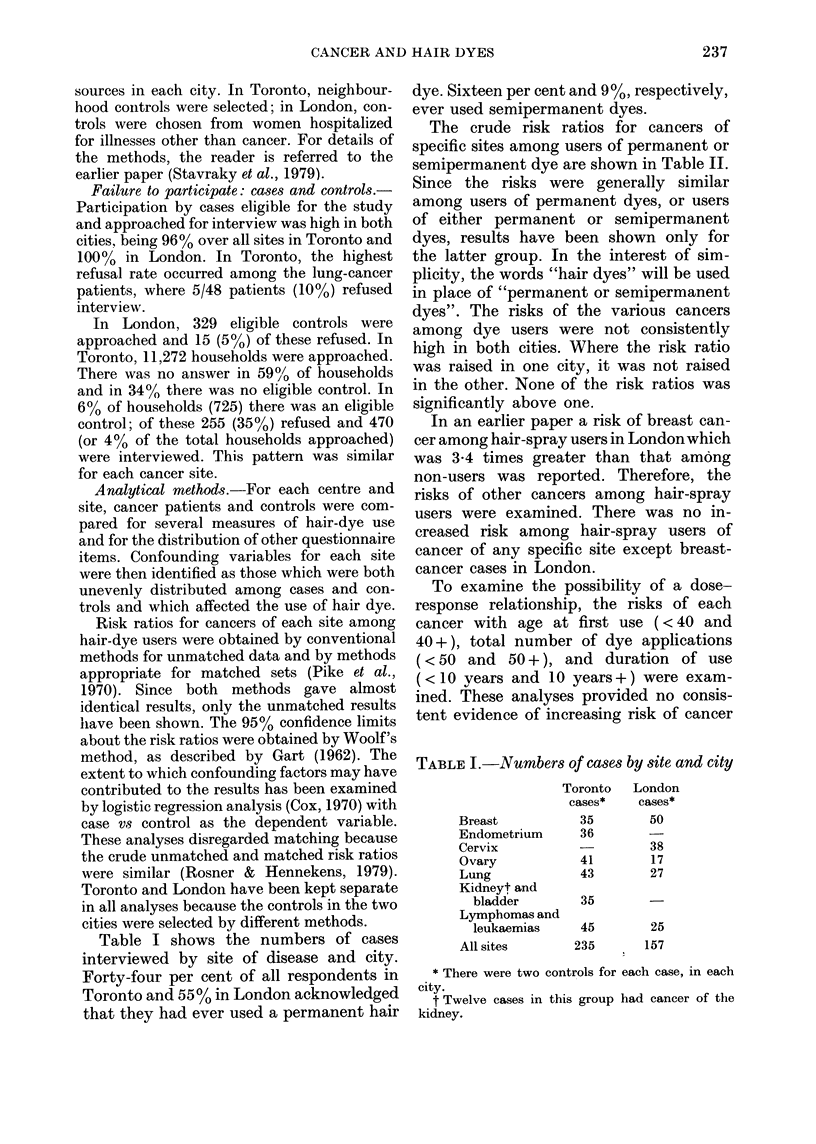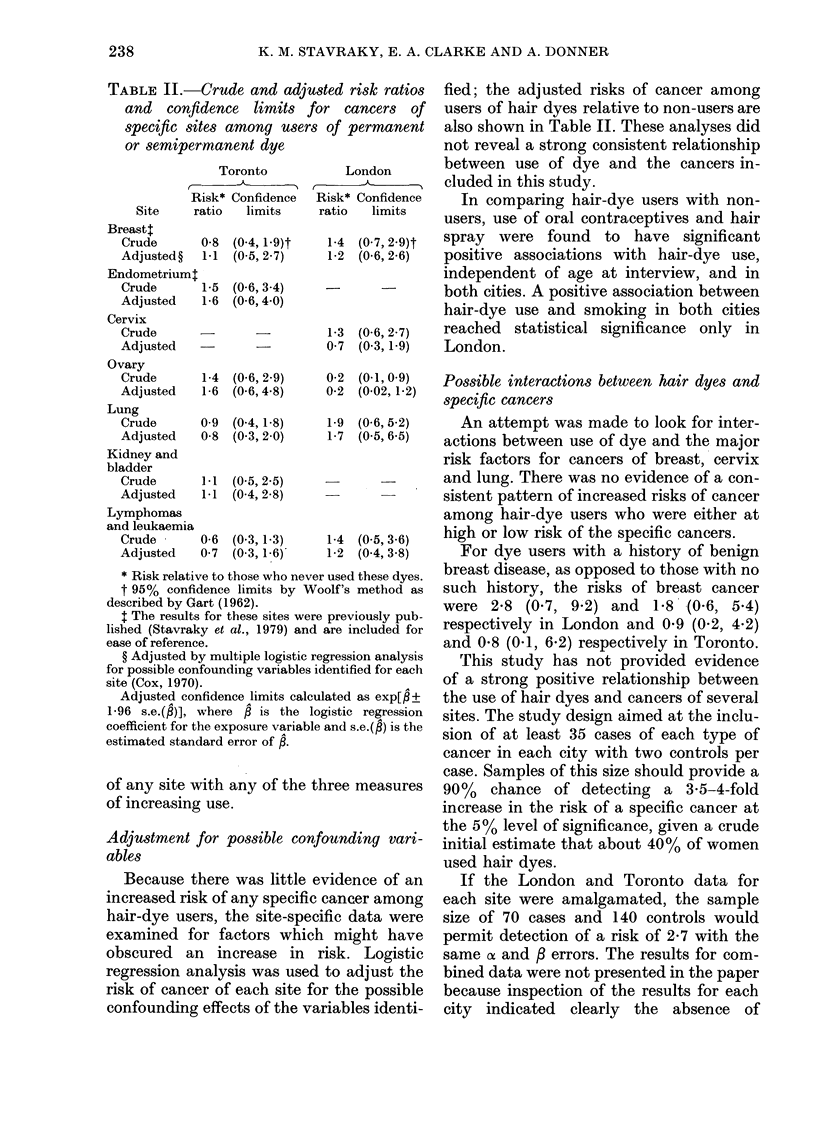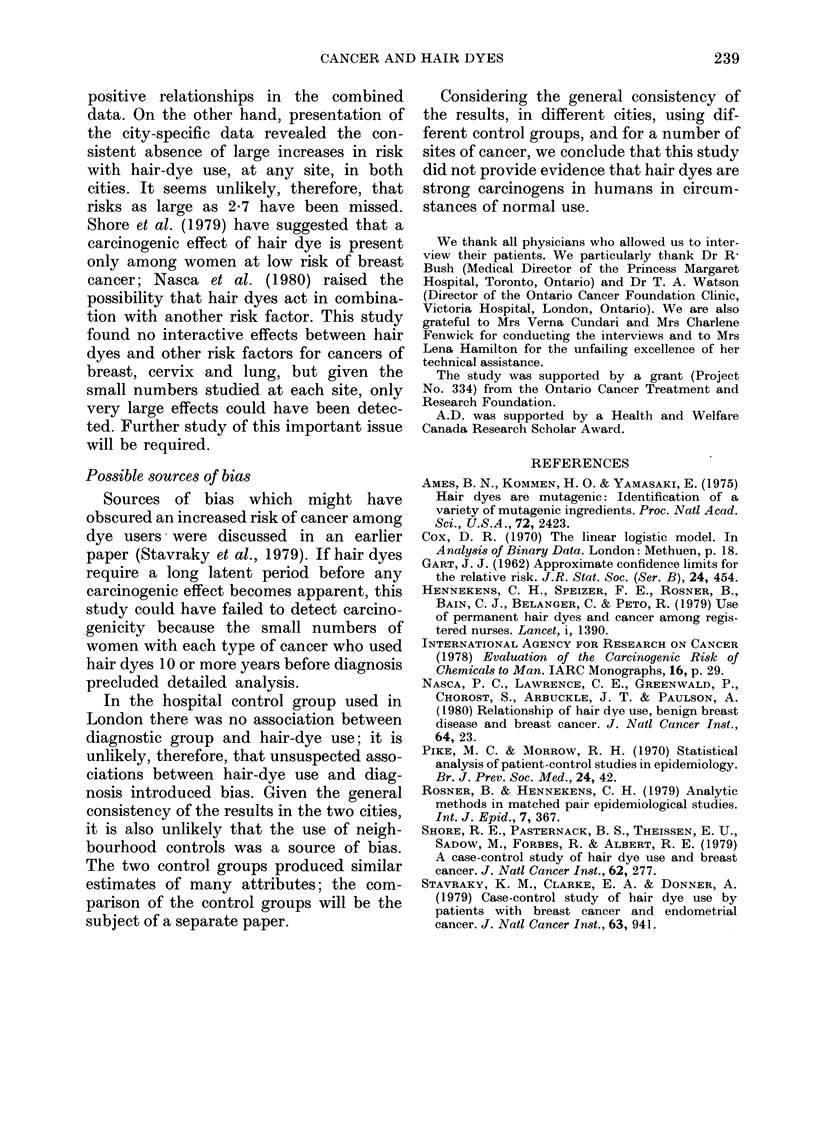# A case-control study of hair-dye use and cancers of various sites.

**DOI:** 10.1038/bjc.1981.35

**Published:** 1981-02

**Authors:** K. M. Stavraky, E. A. Clarke, A. Donner


*Br. J. Cancer* (1981) **43**, 236

***Short Communication***

### A CASE-CONTROL STUDY OF HAIR-DYE USE AND CANCERS OF VARIOUS SITES

K. M. STAVRAKY*, E. A. CLARKE† AND A. DONNER*

*From the *Department of Epidemiology and Biostatistics, University of Western Ontario, London, Ontario, and †Epidemiology and Medical Statistics Unit, OCTRF, McMurrich Building, University of Toronto, Toronto, Canada*

Received 2 July 1980 Accepted 25 October 1980

IN 1975, Ames and his colleagues showed that permanent and semipermanent hair dyes and some of their constituents were mutagenic in a bacterial screening system (Ames *et al.*, 1975). Following this work, a number of epidemiological studies of the carcinogenicity of hair dyes have been reported. This literature was summarized in an earlier report of this study (Stavraky *et al.*, 1979), and in an IARC monograph (IARC, 1978). Since these publications, three epidemiological studies have been reported. Shore *et al.* (1979), in a study of 129 breast-cancer patients and 193 controls drawn from a multiphasic screening clinic, showed a statistically significant relationship between quantity of dye used (number of years used multiplied by annual frequency) and breast cancer after controlling for confounding variables. The relationship was not strong and was virtually confined to women over 50 years of age, and to those at lowest natural risk for breast cancer. Another case-control study by Nasca *et al.* (1980) of 118 breast cancer patients and 233 controls found no overall association between hair dyes and risk of breast cancer, but a statistically significant risk of 4·5 among women with benign breast disease and exposure to dyes. The risk appeared to be confined to women aged 40–49 at diagnosis.

Hennekens *et al.* (1979) surveyed over 120,000 married female registered nurses between the ages of 30 and 55. They found a 10% increase in risk of cancers of all sites among hair-dye users. Of the individual sites, increased risks were found for cancer of the cervix and vagina and vulva. Adjustment for cigarette smoking reduced the magnitude of the risks. There was no convincing evidence of a steady increase in risk with increasing lapse of time since diagnosis.

Evidence from epidemiological studies that hair dyes are carcinogenic is weak, in part because it is contradictory. Reported here is a case-control study of hair-dye use among women with cancers of several sites, designed to rule as unlikely large increases in the risk of cancer of selected sites among users of permanent or semipermanent dye.

Beginning in June 1976, women with newly diagnosed ($\leqslant 6$ months before admission) cancers of breast, ovary, lung and the lymphomas and leukaemias were identified for interview from all women with these cancers admitted to the Princess Margaret Hospital Out-Patient Clinics, Toronto, Ontario, and the Ontario Cancer Foundation Clinic, Victoria Hospital, London, Ontario.

Endometrial and bladder-cancer cases were identified in Toronto only, and cervical cancer cases in London only, from the same clinic

Correspondence to: Dr K. M. Stavraky, Department of Epidemiology and Biostatistics, University of Western Ontario, London, Ontario, Canada N6A 5B7.

sources in each city. In Toronto, neighbourhood controls were selected; in London, controls were chosen from women hospitalized for illnesses other than cancer. For details of the methods, the reader is referred to the earlier paper (Stavraky *et al.*, 1979).

*Failure to participate: cases and controls.*—Participation by cases eligible for the study and approached for interview was high in both cities, being 96% over all sites in Toronto and 100% in London. In Toronto, the highest refusal rate occurred among the lung-cancer patients, where 5/48 patients (10%) refused interview.

In London, 329 eligible controls were approached and 15 (5%) of these refused. In Toronto, 11,272 households were approached. There was no answer in 59% of households and in 34% there was no eligible control. In 6% of households (725) there was an eligible control; of these 255 (35%) refused and 470 (or 4% of the total households approached) were interviewed. This pattern was similar for each cancer site.

*Analytical methods.*—For each centre and site, cancer patients and controls were compared for several measures of hair-dye use and for the distribution of other questionnaire items. Confounding variables for each site were then identified as those which were both unevenly distributed among cases and controls and which affected the use of hair dye.

Risk ratios for cancers of each site among hair-dye users were obtained by conventional methods for unmatched data and by methods appropriate for matched sets (Pike *et al.*, 1970). Since both methods gave almost identical results, only the unmatched results have been shown. The 95% confidence limits about the risk ratios were obtained by Woolf's method, as described by Gart (1962). The extent to which confounding factors may have contributed to the results has been examined by logistic regression analysis (Cox, 1970) with case *vs* control as the dependent variable. These analyses disregarded matching because the crude unmatched and matched risk ratios were similar (Rosner & Hennekens, 1979). Toronto and London have been kept separate in all analyses because the controls in the two cities were selected by different methods.

Table I shows the numbers of cases interviewed by site of disease and city. Forty-four per cent of all respondents in Toronto and 55% in London acknowledged that they had ever used a permanent hair dye. Sixteen per cent and 9%, respectively, ever used semipermanent dyes.

The crude risk ratios for cancers of specific sites among users of permanent or semipermanent dye are shown in Table II. Since the risks were generally similar among users of permanent dyes, or users of either permanent or semipermanent dyes, results have been shown only for the latter group. In the interest of simplicity, the words "hair dyes" will be used in place of "permanent or semipermanent dyes". The risks of the various cancers among dye users were not consistently high in both cities. Where the risk ratio was raised in one city, it was not raised in the other. None of the risk ratios was significantly above one.

In an earlier paper a risk of breast cancer among hair-spray users in London which was 3·4 times greater than that among non-users was reported. Therefore, the risks of other cancers among hair-spray users were examined. There was no increased risk among hair-spray users of cancer of any specific site except breast-cancer cases in London.

To examine the possibility of a dose–response relationship, the risks of each cancer with age at first use (<40 and 40+), total number of dye applications (<50 and 50+), and duration of use (<10 years and 10 years+) were examined. These analyses provided no consistent evidence of increasing risk of cancer

TABLE I.—*Numbers of cases by site and city*

| | Toronto cases* | London cases* |
|---|---|---|
| Breast | 35 | 50 |
| Endometrium | 36 | — |
| Cervix | — | 38 |
| Ovary | 41 | 17 |
| Lung | 43 | 27 |
| Kidney† and bladder | 35 | — |
| Lymphomas and leukaemias | 45 | 25 |
| All sites | 235 | 157 |

* There were two controls for each case, in each city.

† Twelve cases in this group had cancer of the kidney.

TABLE II.—*Crude and adjusted risk ratios and confidence limits for cancers of specific sites among users of permanent or semipermanent dye*

| | Toronto | | London | |
|---|---|---|---|---|
| Site | Risk* ratio | Confidence limits | Risk* ratio | Confidence limits |
| Breast‡ | | | | |
| Crude | 0·8 | (0·4, 1·9)† | 1·4 | (0·7, 2·9)† |
| Adjusted§ | 1·1 | (0·5, 2·7) | 1·2 | (0·6, 2·6) |
| Endometrium‡ | | | | |
| Crude | 1·5 | (0·6, 3·4) | — | — |
| Adjusted | 1·6 | (0·6, 4·0) | | |
| Cervix | | | | |
| Crude | — | — | 1·3 | (0·6, 2·7) |
| Adjusted | — | — | 0·7 | (0·3, 1·9) |
| Ovary | | | | |
| Crude | 1·4 | (0·6, 2·9) | 0·2 | (0·1, 0·9) |
| Adjusted | 1·6 | (0·6, 4·8) | 0·2 | (0·02, 1·2) |
| Lung | | | | |
| Crude | 0·9 | (0·4, 1·8) | 1·9 | (0·6, 5·2) |
| Adjusted | 0·8 | (0·3, 2·0) | 1·7 | (0·5, 6·5) |
| Kidney and bladder | | | | |
| Crude | 1·1 | (0·5, 2·5) | — | — |
| Adjusted | 1·1 | (0·4, 2·8) | — | — |
| Lymphomas and leukaemia | | | | |
| Crude | 0·6 | (0·3, 1·3) | 1·4 | (0·5, 3·6) |
| Adjusted | 0·7 | (0·3, 1·6) | 1·2 | (0·4, 3·8) |

* Risk relative to those who never used these dyes.

† 95% confidence limits by Woolf's method as described by Gart (1962).

‡ The results for these sites were previously published (Stavraky *et al.*, 1979) and are included for ease of reference.

§ Adjusted by multiple logistic regression analysis for possible confounding variables identified for each site (Cox, 1970).

Adjusted confidence limits calculated as $\exp[\hat{\beta} \pm 1{\cdot}96\ \text{s.e.}(\hat{\beta})]$, where $\hat{\beta}$ is the logistic regression coefficient for the exposure variable and s.e.($\hat{\beta}$) is the estimated standard error of $\hat{\beta}$.

of any site with any of the three measures of increasing use.

*Adjustment for possible confounding variables*

Because there was little evidence of an increased risk of any specific cancer among hair-dye users, the site-specific data were examined for factors which might have obscured an increase in risk. Logistic regression analysis was used to adjust the risk of cancer of each site for the possible confounding effects of the variables identified; the adjusted risks of cancer among users of hair dyes relative to non-users are also shown in Table II. These analyses did not reveal a strong consistent relationship between use of dye and the cancers included in this study.

In comparing hair-dye users with non-users, use of oral contraceptives and hair spray were found to have significant positive associations with hair-dye use, independent of age at interview, and in both cities. A positive association between hair-dye use and smoking in both cities reached statistical significance only in London.

*Possible interactions between hair dyes and specific cancers*

An attempt was made to look for interactions between use of dye and the major risk factors for cancers of breast, cervix and lung. There was no evidence of a consistent pattern of increased risks of cancer among hair-dye users who were either at high or low risk of the specific cancers.

For dye users with a history of benign breast disease, as opposed to those with no such history, the risks of breast cancer were 2·8 (0·7, 9·2) and 1·8 (0·6, 5·4) respectively in London and 0·9 (0·2, 4·2) and 0·8 (0·1, 6·2) respectively in Toronto.

This study has not provided evidence of a strong positive relationship between the use of hair dyes and cancers of several sites. The study design aimed at the inclusion of at least 35 cases of each type of cancer in each city with two controls per case. Samples of this size should provide a 90% chance of detecting a 3·5–4-fold increase in the risk of a specific cancer at the 5% level of significance, given a crude initial estimate that about 40% of women used hair dyes.

If the London and Toronto data for each site were amalgamated, the sample size of 70 cases and 140 controls would permit detection of a risk of 2·7 with the same $\alpha$ and $\beta$ errors. The results for combined data were not presented in the paper because inspection of the results for each city indicated clearly the absence of positive relationships in the combined data. On the other hand, presentation of the city-specific data revealed the consistent absence of large increases in risk with hair-dye use, at any site, in both cities. It seems unlikely, therefore, that risks as large as 2·7 have been missed. Shore *et al.* (1979) have suggested that a carcinogenic effect of hair dye is present only among women at low risk of breast cancer; Nasca *et al.* (1980) raised the possibility that hair dyes act in combination with another risk factor. This study found no interactive effects between hair dyes and other risk factors for cancers of breast, cervix and lung, but given the small numbers studied at each site, only very large effects could have been detected. Further study of this important issue will be required.

### *Possible sources of bias*

Sources of bias which might have obscured an increased risk of cancer among dye users were discussed in an earlier paper (Stavraky *et al.*, 1979). If hair dyes require a long latent period before any carcinogenic effect becomes apparent, this study could have failed to detect carcinogenicity because the small numbers of women with each type of cancer who used hair dyes 10 or more years before diagnosis precluded detailed analysis.

In the hospital control group used in London there was no association between diagnostic group and hair-dye use; it is unlikely, therefore, that unsuspected associations between hair-dye use and diagnosis introduced bias. Given the general consistency of the results in the two cities, it is also unlikely that the use of neighbourhood controls was a source of bias. The two control groups produced similar estimates of many attributes; the comparison of the control groups will be the subject of a separate paper.

Considering the general consistency of the results, in different cities, using different control groups, and for a number of sites of cancer, we conclude that this study did not provide evidence that hair dyes are strong carcinogens in humans in circumstances of normal use.

We thank all physicians who allowed us to interview their patients. We particularly thank Dr R· Bush (Medical Director of the Princess Margaret Hospital, Toronto, Ontario) and Dr T. A. Watson (Director of the Ontario Cancer Foundation Clinic, Victoria Hospital, London, Ontario). We are also grateful to Mrs Verna Cundari and Mrs Charlene Fenwick for conducting the interviews and to Mrs Lena Hamilton for the unfailing excellence of her technical assistance.

The study was supported by a grant (Project No. 334) from the Ontario Cancer Treatment and Research Foundation.

A.D. was supported by a Health and Welfare Canada Research Scholar Award.

### REFERENCES

AMES, B. N., KOMMEN, H. O. & YAMASAKI, E. (1975) Hair dyes are mutagenic: Identification of a variety of mutagenic ingredients. *Proc. Natl Acad. Sci., U.S.A.*, **72**, 2423.

COX, D. R. (1970) The linear logistic model. In *Analysis of Binary Data*. London: Methuen, p. 18.

GART, J. J. (1962) Approximate confidence limits for the relative risk. *J.R. Stat. Soc. (Ser. B)*, **24**, 454.

HENNEKENS, C. H., SPEIZER, F. E., ROSNER, B., BAIN, C. J., BELANGER, C. & PETO, R. (1979) Use of permanent hair dyes and cancer among registered nurses. *Lancet*, i, 1390.

INTERNATIONAL AGENCY FOR RESEARCH ON CANCER (1978) *Evaluation of the Carcinogenic Risk of Chemicals to Man.* IARC Monographs, **16**, p. 29.

NASCA, P. C., LAWRENCE, C. E., GREENWALD, P., CHOROST, S., ARBUCKLE, J. T. & PAULSON, A. (1980) Relationship of hair dye use, benign breast disease and breast cancer. *J. Natl Cancer Inst.*, **64**, 23.

PIKE, M. C. & MORROW, R. H. (1970) Statistical analysis of patient-control studies in epidemiology. *Br. J. Prev. Soc. Med.*, **24**, 42.

ROSNER, B. & HENNEKENS, C. H. (1979) Analytic methods in matched pair epidemiological studies. *Int. J. Epid.*, **7**, 367.

SHORE, R. E., PASTERNACK, B. S., THEISSEN, E. U., SADOW, M., FORBES, R. & ALBERT, R. E. (1979) A case-control study of hair dye use and breast cancer. *J. Natl Cancer Inst.*, **62**, 277.

STAVRAKY, K. M., CLARKE, E. A. & DONNER, A. (1979) Case-control study of hair dye use by patients with breast cancer and endometrial cancer. *J. Natl Cancer Inst.*, **63**, 941.